# Polygenic Risk Score for Adult Idiopathic Hydrocele Testis: Susceptibility and Severity in a Japanese Cohort

**DOI:** 10.3390/ijms27167143

**Published:** 2026-08-09

**Authors:** Mami Hattori-Kato, Yumiko Okuno, Sachi Honda, Akira Nomiya, Masayoshi Zaitsu, Takumi Takeuchi

**Affiliations:** 1 Department of Urology, Japan Organization of Occupational Health and Safety, Kanto Rosai Hospital, 1-1 Kizukisumiyoshi-cho, Nakahara-ku, Kawasaki 211-8510, Japan; mami_rainbow_rejoice@yahoo.co.jp (M.H.-K.); yumiyumix@hotmail.com (Y.O.); yama03sachi13@gmail.com (S.H.); akira.nomiya@gmail.com (A.N.); 2 Center for Research of the Aging Workforce, University of Occupational and Environmental Health, Fukuoka 807-8556, Japan; mzaitsu@med.uoeh-u.ac.jp

**Keywords:** testicular hydrocele, polygenic risk score, single nucleotide polymorphism, genome-wide association study, disease susceptibility, disease severity, East Asian population, cross-population transferability

## Abstract

This exploratory study evaluated whether a polygenic risk score (PRS) derived from a European genome-wide association study is transferable to a Japanese cohort, for both susceptibility to and severity of adult idiopathic hydrocele testis. Surgically confirmed hydrocele cases were compared with two independently ascertained control groups: prostate cancer patients with intraoperatively confirmed absence of hydrocele fluid (Control 1), and urolithiasis patients without malignancy (Stone Control). Multivariable logistic regression evaluated disease occurrence; multiple linear regression restricted to hydrocele cases evaluated the association between PRS and fluid volume. A higher PRS was associated with increased susceptibility to hydrocele when cases were compared with Stone Controls (strictest threshold: odds ratio 1.98, 95% CI 1.01–3.88, *p* = 0.048; AUC 0.733), though this was not observed using Control 1. Among cases, PRS showed a consistent inverse association with fluid volume across all three thresholds tested (standardized β = −0.54, *p* = 0.010 at ≥10 mL). Given the modest sample size and limited statistical power, these findings should be regarded as preliminary and hypothesis-generating. They nonetheless suggest that genetic susceptibility to hydrocele and its severity may show different associations with polygenic risk, a pattern requiring confirmation in larger, independent, ideally East Asian-derived cohorts.

## 1. Introduction

Hydrocele testis is a common benign condition characterized by the excessive accumulation of serous fluid between the parietal and visceral layers of the tunica vaginalis, resulting in scrotal swelling. Based on its etiology, hydrocele is broadly classified as primary (idiopathic) or secondary to an identifiable cause such as infection, trauma, surgery, or malignancy [1]. Within the primary group, “communicating” hydroceles are predominantly observed in children and are caused by a patent processus vaginalis, whereas “non-communicating” hydroceles typically occur in adults and are thought to arise from an imbalance between the secretion of serous fluid within the tunica vaginalis and its absorption via the lymphatic and venous systems. The majority of adult hydroceles are idiopathic, without any identifiable preceding factor. Idiopathic hydroceles cause physical discomfort and cosmetic concern due to progressive scrotal enlargement, often necessitating surgery. The fundamental etiology and pathophysiology of this fluid imbalance in adult, non-communicating, idiopathic hydrocele nevertheless remain unclear.

We have continuously investigated the pathophysiology of adult non-communicating idiopathic hydrocele from a molecular biological perspective. In our initial study, aquaporin 1 (AQP1), a water channel protein [2], was found to be significantly overexpressed in the capillary endothelial cells of the tunica vaginalis in hydrocele patients, suggesting its involvement in disease pathogenesis [3]. In a subsequent epigenetic analysis, we further showed that DNA hypomethylation at a specific CpG site within the *AQP1* CpG island is associated with upregulation of AQP1 protein expression, and may contribute to the formation of adult-onset non-communicating hydrocele testis [4].

While hydrocele has historically been considered primarily a local anatomical or molecular abnormality, Roberson et al. recently reported a genome-wide association study (GWAS) of adult hydrocele in large-scale cohorts (UK Biobank and FinnGen) [5]. That study identified seven genome-wide significant loci, mapping to 24 genes—including *PAX8*, *INHBB*, *AMHR2*, and *SHH*—with roles in genitourinary embryogenesis, providing the first evidence for a genetic contribution to adult hydrocele. However, the associated SNPs were identified predominantly in populations of European ancestry. Because allele frequencies and linkage disequilibrium (LD) structure vary across ethnic groups, whether these markers are directly applicable to a Japanese population is unknown.

A polygenic risk score (PRS) summarizes an individual’s overall genetic predisposition to a multifactorial disease by aggregating the small effect sizes of numerous associated SNPs [6,7,8]. In a cohort of limited size, an external PRS—applying effect sizes from an independent, larger discovery GWAS—is generally considered preferable to an internally derived PRS, since internal weighting in small samples is prone to overfitting. This preference, however, depends on several factors, including ancestry matching between discovery and target cohorts and the precision of phenotype ascertainment, and should not be regarded as absolute [8]. Applying a PRS derived from a European GWAS to another population can also result in attenuated predictive accuracy owing to ancestral differences [6,9], making direct examination of cross-population transferability important.

In the present study, we calculated an external PRS using the adult-hydrocele-associated SNPs identified by Roberson et al. [5] to examine (i) whether this score predicts the occurrence of adult, non-communicating, idiopathic hydrocele testis, and (ii) whether it is associated with hydrocele fluid volume, in a Japanese cohort.

## 2. Results

**Participant characteristics**

**Figure 1:**
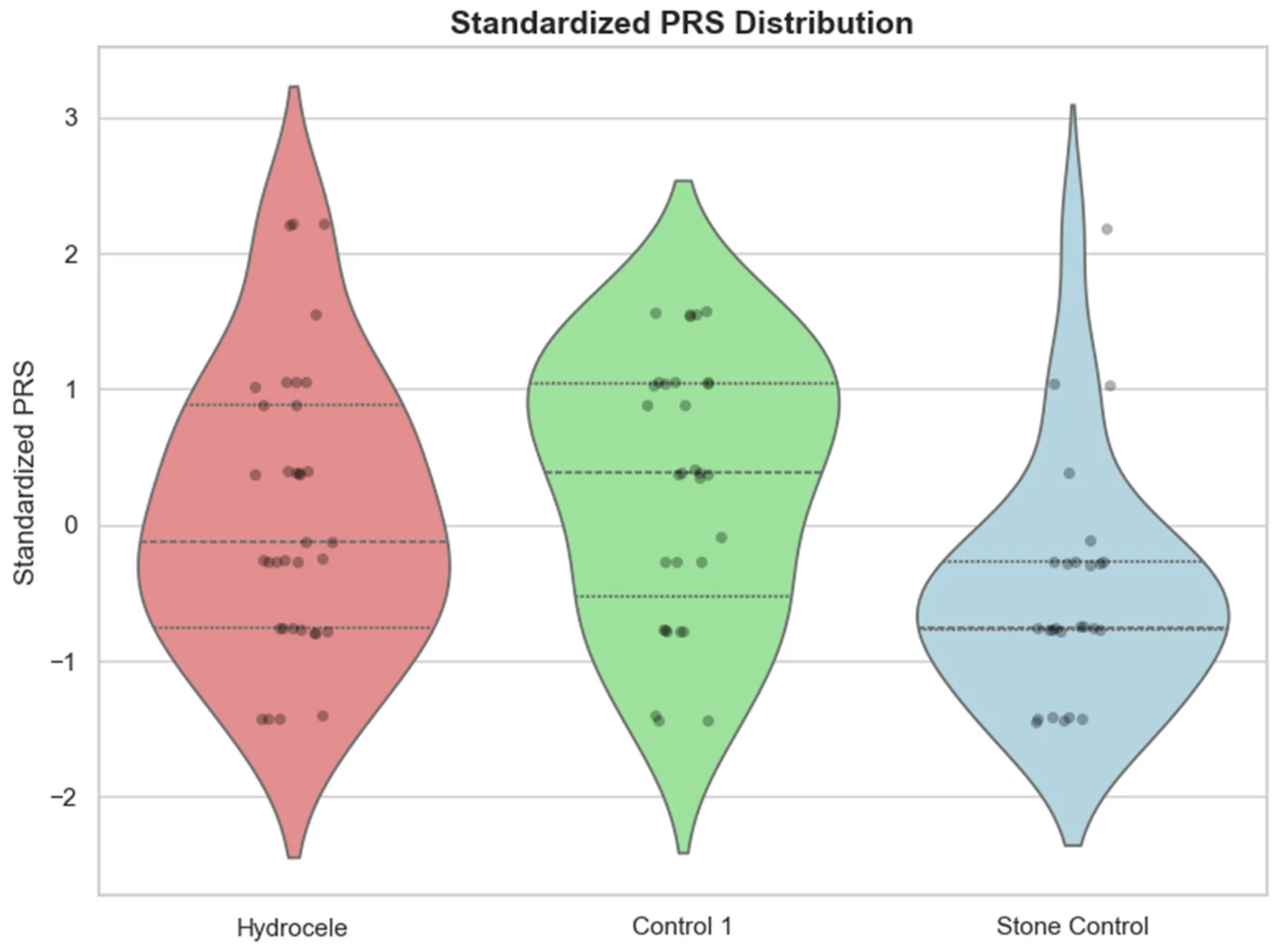
Standardized polygenic risk score (PRS) distribution by group, shown as violin plots with individual data points overlaid. Hydrocele: Surgically confirmed idiopathic hydrocele cases (n = 35 with genotype data). Control 1: Prostate cancer patients who underwent bilateral orchiectomy, with hydrocele fluid absence confirmed intraoperatively (n = 31). Stone Control: Urolithiasis patients without prior malignancy or hydrocele (n = 26). The PRS was standardized (mean 0, SD 1) across the combined sample. Horizontal dashed lines within each violin indicate the median and interquartile range. The PRS was significantly lower in Stone Control than in Control 1 (*p* = 0.0002), and did not differ significantly between Hydrocele and either control group individually (Table 1); possible interpretations are discussed in the main text.

Participant characteristics are summarized in Table 1. The number of subjects varies slightly across specific analyses depending on the availability of fluid-volume records or DNA samples. Hydrocele fluid volume had a median of 66 mL (range 1.1–510 mL, n = 40); for bilateral cases, the volume of the larger side was used. The standardized PRS was significantly lower in the Stone Control group than in the Control 1 (prostate cancer) group (*p* = 0.0002, Figure 1); possible interpretations of this difference are discussed below.

### 2.1. Prediction of Hydrocele Testis Susceptibility

Base clinical models (age, Brinkman index, drinking history, occupation) were compared with full models additionally incorporating PRS, across control groups and phenotypic thresholds. Using Control 1, adding PRS to the base model did not significantly improve discrimination (AUC 0.671 to 0.696; DeLong’s test *p* = 0.432), and PRS was not an independent predictor of hydrocele (OR = 0.78, 95% CI 0.49–1.25, *p* = 0.309) (Table 2).

**Figure 2:**
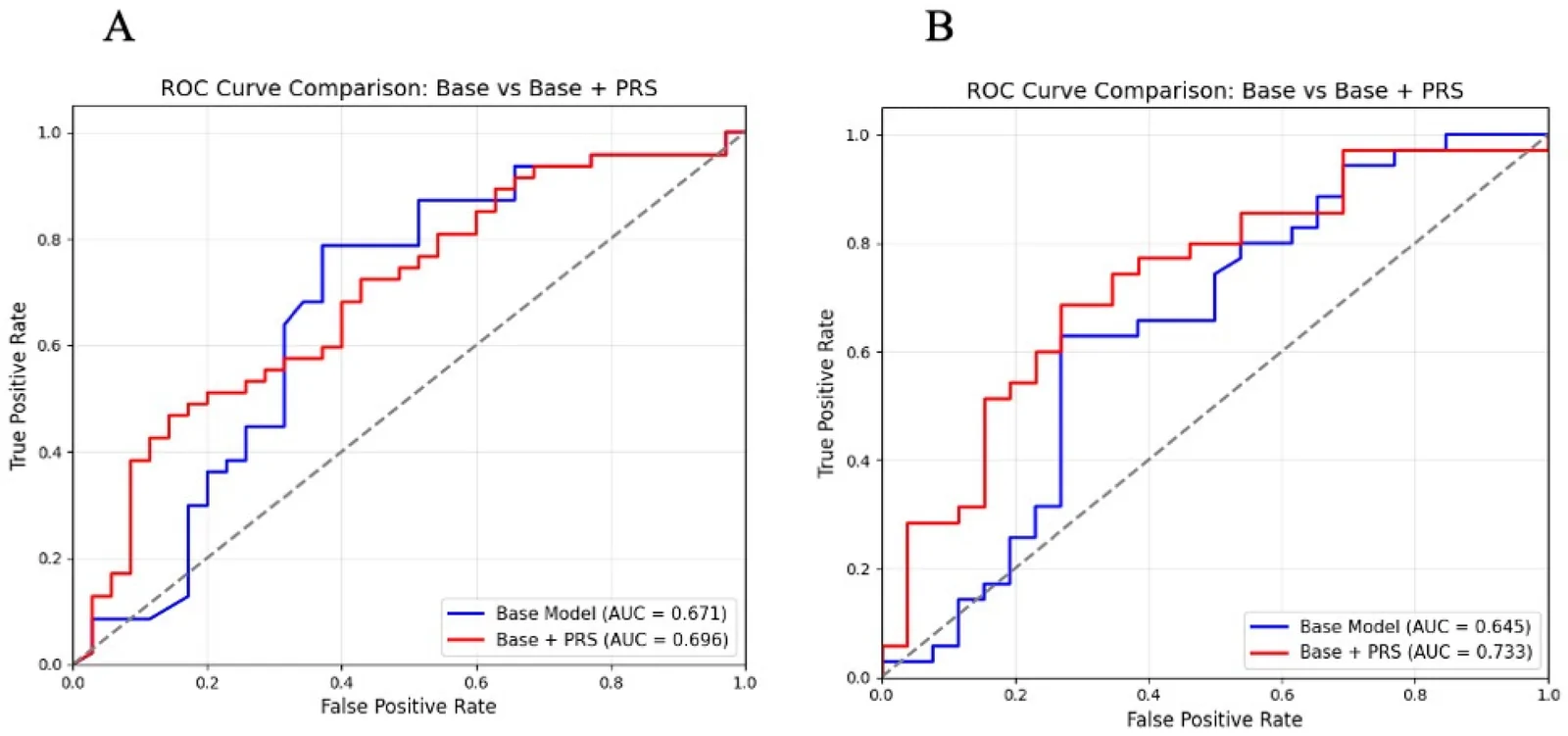
Receiver operating characteristic (ROC) curves for prediction of hydrocele testis occurrence, comparing a base clinical model (age, Brinkman index, drinking history, occupation; blue line) with a full model additionally including PRS (red line). The gray diagonal represents chance discrimination (AUC = 0.5). (**A**) Hydrocele cases with fluid volume > 1 mL versus Control 1 (base AUC = 0.671, full AUC = 0.696; DeLong’s test *p* = 0.432). (**B**) Hydrocele cases with fluid volume ≥ 10 mL versus Stone Control (base AUC = 0.645, full AUC = 0.733; DeLong’s test *p* = 0.154). Numerical results for all four control-group/threshold combinations are given in Table 3.

In contrast, when hydrocele cases were compared with the Stone Control group, PRS showed a significant, positive association with disease susceptibility across all three fluid-volume thresholds (Table 2, Table 3). In the most restrictive comparison (≥10 mL vs. Stone Control), a 1-SD increase in PRS was associated with an odds ratio of 1.98 (95% CI 1.01–3.88, *p* = 0.048), and the full model achieved an AUC of 0.733 (Figure 2), representing moderate rather than strong discrimination [10]. Several possible, non-mutually exclusive explanations for this discrepancy between the two control groups—including the possibility that it reflects a property of the Stone Control group itself rather than a hydrocele-specific effect—are considered in the Discussion; we present both comparisons transparently rather than treating one as definitive.

### 2.2. Association Between PRS and Hydrocele Fluid Volume

Hydrocele fluid volume is, by definition, undefined in individuals without hydrocele. Because PRS was itself associated with case status in the Stone Control comparison (above), a regression that assigns controls a fixed volume of 0 mL and analyzes cases and controls jointly conflates the genetic determinants of disease occurrence with those of disease severity (see Discussion for a direct empirical demonstration of this). We therefore restricted the primary severity analysis to hydrocele cases only, separately for each of the three case-inclusion thresholds. Because this analysis involves no control group, its results do not depend on which control group was used for the susceptibility analysis above (Table 4).

**Figure 3:**
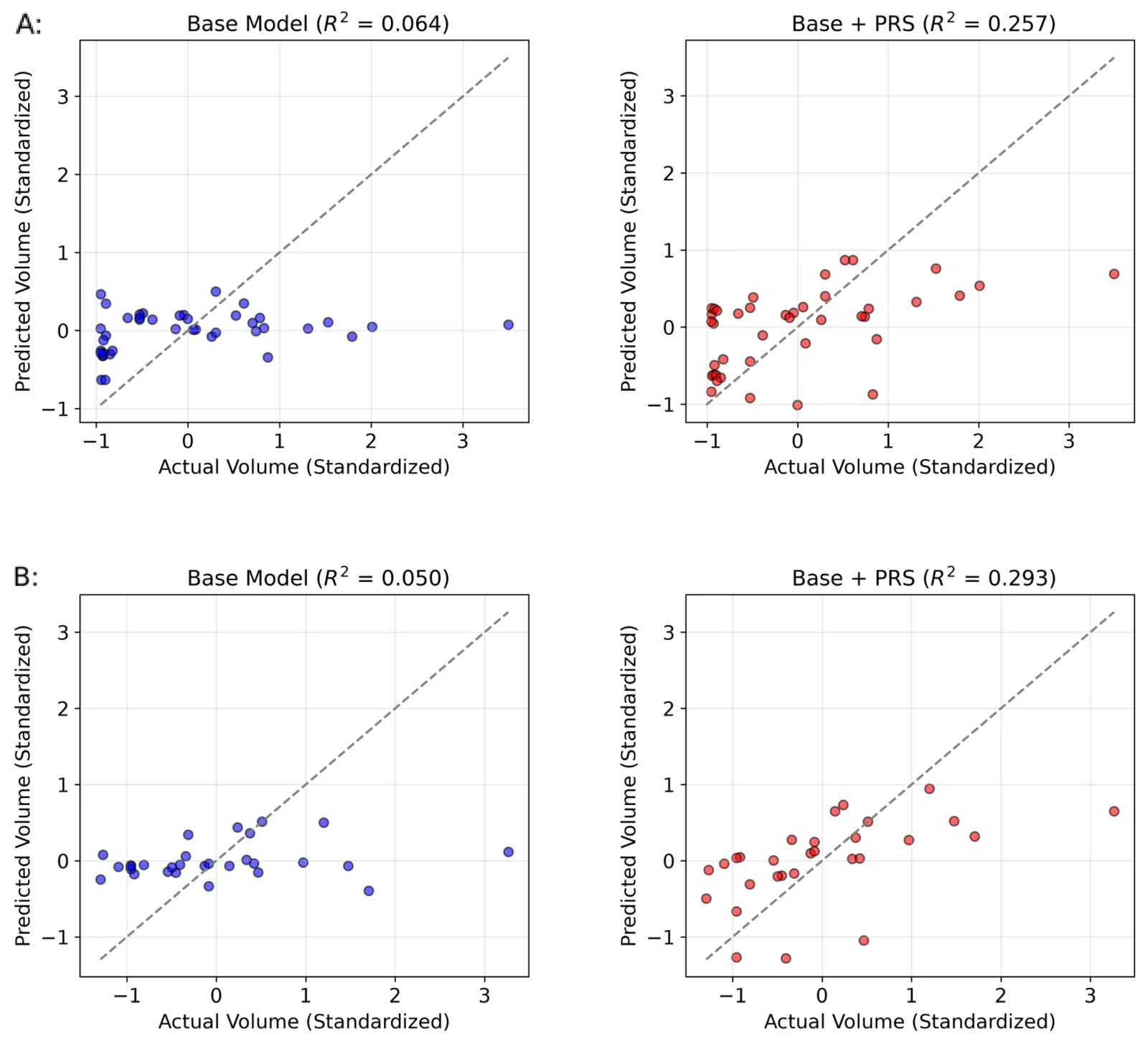
Scatter plots of actual versus model-predicted, standardized hydrocele fluid volume, restricted to hydrocele cases (no control subjects included), comparing a base clinical model (left, blue points) with a full model additionally including PRS (right, red points). The gray diagonal line represents perfect prediction (predicted = actual). (**A**) All hydrocele cases with recorded volume (>1 mL threshold, n = 41): base R^2^ = 0.064, full R^2^ = 0.257. (**B**) Hydrocele cases with volume ≥ 10 mL (n = 29): base R^2^ = 0.050, full R^2^ = 0.293. Corresponding regression statistics, including the PRS coefficient and ANOVA comparison of the base and full models, are given in Table 5. This figure replaces the version in the previous submission, which included Control 1 or Stone Control subjects fixed at a volume of 0 mL and no longer corresponded to the case-only models reported in the revised results.

Among hydrocele cases, PRS showed a consistent inverse association with fluid volume across all three thresholds (Table 5). At the strictest threshold (≥ 10 mL), the full model explained 29.3% of the variance in standardized fluid volume (R^2^ = 0.293, ANOVA *p* = 0.010), and PRS remained an independent, inverse predictor of volume (standardized β = −0.536, *p* = 0.010; Figure 3). We interpret this as preliminary evidence that, among individuals who develop hydrocele, a higher genetic risk burden is associated with a smaller fluid volume; possible biological explanations, and important caveats, are discussed below.

## 3. Discussion

In this exploratory study, we examined the cross-population transferability of a European GWAS-derived PRS for adult hydrocele testis in a Japanese cohort, and its relationship to both disease occurrence and hydrocele fluid volume. Two observations stood out, and we discuss each with the caution warranted by our sample size.

### 3.1. Divergent Results Between the Two Control Groups

PRS was not significantly associated with hydrocele occurrence when Control 1 (prostate cancer patients) was used as the reference group, but was significantly and positively associated with occurrence when the Stone Control group was used. One possible explanation is pleiotropic enrichment of hydrocele-associated variants in the prostate cancer group: both PAX8 and SHH have documented roles in prostate tumorigenesis [11,12], which could elevate baseline PRS in Control 1 and obscure the association with hydrocele. This is consistent with the significantly higher PRS observed in Control 1 relative to Stone Control (Table 1, Figure 1). An alternative, and equally plausible, explanation is that one or more of the loci in our PRS are independently associated with urolithiasis risk, so that the lower PRS in the Stone Control group reflects a genuine relationship with stone disease rather than, or in addition to, a specific relationship with hydrocele. In particular, INHBB, one of the five loci included in our PRS, has been reported as a serum urate-associated locus in a large genome-wide association study [13], and elevated serum urate is an established risk factor for urate stone formation. We did not find comparable associations for *PAX8*, *SHH*, *ZNF438*, or *AMHR2* in the urolithiasis or serum urate genetics literature we reviewed, so we do not think this fully accounts for the pattern observed with the full five-SNP score, but a partial contribution cannot be excluded. We were unable to identify a third, general-population or disease-free control group in the present dataset that would allow this question to be resolved directly, and we regard it as an important target for future validation rather than a settled matter. Neither control group is ideal: Control 1 comprises men with a different, and potentially pleiotropically related, malignancy, while Stone Control comprises men with a different urological condition whose absence of hydrocele was ascertained by physical examination and history rather than by routine scrotal ultrasonography, so a degree of misclassification (inclusion of men with small, undetected hydroceles) cannot be excluded. A formal sensitivity analysis to quantify this possibility was not possible, because ultrasonographic data were not collected in the Stone Control group; we flag this as an unresolved limitation rather than one we can bound quantitatively. This kind of ascertainment difficulty—in which the choice and definition of a comparison group can itself shape the genetic association observed—is a recognized methodological challenge in case–control genetic association research more broadly, and is not unique to this study [14].

### 3.2. Rationale for Restricting the Severity Analysis to Hydrocele Cases

For the severity analysis, we restricted the regression to hydrocele cases rather than assigning controls a fixed fluid volume of 0 mL and analyzing cases and controls jointly. This choice reflects both a conceptual and an empirical consideration. Conceptually, when case status is itself associated with PRS, as observed for the Stone Control comparison, a joint case/control regression combines two effects—the association of PRS with becoming a case (positive, in our data) and the association of PRS with volume among cases (negative)—that are not readily separable and may partly offset one another. Empirically, we confirmed this directly in a sensitivity analysis: when Stone Control subjects were included in the severity regression at a fixed volume of 0 mL, the inverse PRS–volume association was substantially attenuated (PRS *p*-values rose from 0.004 to 0.010 to approximately 0.36–0.81 across the three thresholds) and was no longer statistically significant at any threshold (data not shown; available from the corresponding author on request). The case-only analysis is not subject to this source of confounding, since it involves no case/control contrast, and its results do not depend on which control group is used for the susceptibility analysis. We regard the case-only results in Table 5 as the primary evidence for a within-case inverse association between PRS and hydrocele volume.

### 3.3. Interpretation: A Preliminary, Hypothesis-Generating Model

Taken together, these findings may be consistent with a model in which genetic and acquired factors contribute differently to hydrocele occurrence versus hydrocele severity. We want to be explicit that this—sometimes summarized as a “dual etiology” idea—is a hypothesis suggested by the data, not a conclusion demonstrated by it: no acquired or environmental exposures were directly measured in this study, and no mechanistic experiments were performed. One speculative reading is that patients with a high PRS carry a congenital microstructural predisposition of the tunica vaginalis that manifests as a smaller, relatively self-limited fluid accumulation, whereas patients with a low PRS who nonetheless develop large hydroceles may do so predominantly through acquired mechanisms, such as age-related lymphatic decline or chronic low-grade inflammation, which would be consistent with our earlier work implicating epigenetic upregulation of AQP1 in fluid accumulation [3,4]. We offer this as a plausible narrative rather than a demonstrated mechanism. We did not assess interactions between PRS and environmental exposures such as smoking or occupation, nor did we collect family-history data on hydrocele or related genitourinary conditions; both would be natural next steps.

### 3.4. Limitations

- The overall sample size was small, and our own power calculation indicated approximately 18% power for the logistic regression and 32% power for the linear regression to detect a typical polygenic effect. Effect sizes as large as those observed here (e.g., OR ≈ 2, R^2^ improvement of roughly 0.19–0.24) are, in a study this size, more likely to be inflated by winner’s-curse-type phenomena than in an adequately powered study, and should be treated as provisional estimates.
- We tested three fluid-volume thresholds (> 1, ≥5, ≥10 mL). These were not pre-registered; they were chosen post hoc to explore robustness to progressively stricter case definitions and to partially address possible misclassification in the Stone Control group. We report results at all three thresholds, and the direction and approximate magnitude of the PRS association were consistent across them, but we did not apply a formal correction for multiple comparisons across the several models reported here, and some individual *p*-values, in particular those closest to 0.05, may represent false positives.
- The AUC of 0.733 achieved by the best-performing model represents moderate, not strong, discriminative performance [10], and this PRS is not, in its current form, suitable for clinical risk stratification.
- The PRS was constructed from a small number of SNPs derived from a European-ancestry GWAS. Three of the seven originally reported loci could not be used as originally reported: *CLU* was monoallelic in our population, *MAPK8* had no genotyped or adequately proxied SNP available on our array, and the *INHBB* lead variant was reported only as a positional identifier and required cross-referencing to an rs-numbered proxy (see Methods). A larger, East Asian-specific GWAS of hydrocele would be needed to construct a properly optimized PRS for this population.
- The Stone Control group’s hydrocele status was ascertained by physical examination and history rather than ultrasonography, and we could not perform a sensitivity analysis to quantify the resulting misclassification risk.

Despite these limitations, the associations we observed were broadly consistent in direction across control groups and thresholds, which is compatible with, though does not prove, a genuine underlying signal. We think the most defensible framing is that this is a hypothesis-generating pilot study: it suggests that an externally derived PRS may retain some predictive value for hydrocele susceptibility in a Japanese cohort, and that PRS may be inversely related to fluid volume among affected individuals, but confirmation in a larger, independent, and preferably East Asian-ancestry-matched cohort is required before either claim can be considered established.

## 4. Conclusions

Our findings are compatible with adult idiopathic hydrocele being a multifactorial condition in which genetic and acquired contributions may act differently on disease onset than on disease severity. This remains a preliminary hypothesis: it is suggested by, not demonstrated by, the present data, and requires confirmation in a larger, independent, and preferably East Asian-ancestry-matched cohort before either the susceptibility or the severity finding can be considered established. More broadly, our experience in this study underscores the importance of careful, transparent control-group selection and of analyzing severity outcomes in a way that does not conflate case/control status with within-case variation, in PRS validation studies of this kind.

## 5. Materials and Methods

The inclusion criteria for the hydrocele group were a surgically confirmed diagnosis of non-communicating idiopathic adult hydrocele at the time of hydrocelectomy. To investigate potential pleiotropic bias and genetic confounding, we established two distinct male control groups. Control 1 consisted predominantly of patients with prostate cancer who underwent bilateral orchiectomy and had visually confirmed absence of hydrocele fluid during surgery. Stone Control consisted of patients diagnosed with urolithiasis who had no history of prostate cancer, other malignancy, or prior scrotal surgery; absence of hydrocele in this group was confirmed by routine physical examination and medical history, but not by ultrasonography (see Limitations). Patients with >0 to ≤1 mL of fluid between the layers of the tunica vaginalis, or evidence of intrascrotal inflammation identified during surgery, were excluded.

Genome-wide genotyping was carried out using the Illumina Infinium Asian Screening Array-24 v1.0 BeadChip (San Diego, CA, USA), performed and called in a blinded manner independent of clinical and phenotypic information. The quality-control pipeline for genomic data, and the interview procedures for lifestyle and occupational history, were performed as previously described [15]. Smoking history (Brinkman index) was classified into four groups (0, 1–399, 400–799, ≥800), drinking history into two levels, and occupation into manual and non-manual labor based on the Japan Standard Occupational Classification, with Construction and Manufacturing sector workers additionally classified as manual labor.

### 5.1. SNP Selection and Biological Rationale

To construct the PRS, we initially considered the seven independent lead SNPs reported by Roberson et al. [5]. Three modifications to this panel were required for our Japanese cohort and genotyping platform. First, the *CLU* locus (lead SNP rs2741346) was excluded because this variant is monoallelic (minor allele frequency = 0) in the East Asian (JPT) population of the 1000 Genomes Project, and is therefore uninformative in our cohort. Second, the *MAPK8* locus (lead SNP rs111302388) was excluded because it was not genotyped on our array and no adequately correlated proxy (r^2^ ≥ 0.8 in East Asian populations) could be identified. Third, the *SHH* lead SNP rs1616634 (7:155653078, GRCh37) was not present on our array and was substituted with rs1731840 (7:155651490), which is in complete linkage disequilibrium with rs1616634 in East Asian populations (r^2^ = 1.0, D’ = 1.0; LDproxy, 1000 Genomes JPT). We confirmed the correspondence between the risk allele of rs1616634 (effect allele C, per the original GWAS) and the linked allele of rs1731840 using NCBI dbSNP reference/alternate allele annotations, and retained the original effect size (β = 0.1409) without inverting its sign. The *INHBB* lead variant reported by Roberson et al. was given only as a positional identifier (2:121125878:G:T, GRCh37) rather than as an rs number. Based on GRCh37 coordinates, this variant corresponds to rs2164725, which is the identifier used in Table 6 and in our array design. Following these three modifications, five SNPs mapping to *PAX8*, *INHBB*, *SHH*, *ZNF438*, and *AMHR2* (rs3748916, rs2164725, rs1731840, rs1087056, and rs11170549, respectively) were used for the final PRS calculation (Table 6), consistent with standard PRS construction practice [16].

### 5.2. Statistical Analysis

To evaluate the predictive value of PRS for hydrocele susceptibility and severity, we conducted multivariable logistic regression and multiple linear regression. To address possible misclassification in the Stone Control group, we performed a phenotypic-refinement analysis using three fluid-volume inclusion thresholds for hydrocele cases: all cases (>1 mL), ≥5 mL, and ≥10 mL.

The thresholds of 1, 5, and 10 mL were not pre-registered; they were selected post hoc to examine the sensitivity of our findings to progressively stricter phenotype definitions. We report results at all three thresholds rather than only the most favorable one, in the interest of transparency, although we recognize this does not by itself rule out selective emphasis in the interpretation of a threshold-varying result.

For the logistic regression evaluating disease susceptibility, the base model included age, Brinkman index category, drinking history, and occupation, while the full model additionally incorporated PRS. Model discrimination was assessed by AUC, and differences between models were tested with DeLong’s test. Missing clinical covariate data were addressed with multiple imputation by chained equations (MICE) prior to model fitting.

For the severity analysis, we performed multiple linear regression (ordinary least squares) using hydrocele fluid volume as the dependent variable, restricted to hydrocele cases only, separately at each of the three case-inclusion thresholds; the rationale for this restriction, and a sensitivity analysis in which controls were instead assigned a fixed volume of 0 mL, are described in the Discussion. Missing covariate data among cases were addressed with MICE, as for the logistic models. Both the dependent variable and continuous/ordinal covariates were standardized (mean 0, SD 1) before analysis, and the improvement in explained variance (R^2^) upon adding PRS was tested with an ANOVA F-test.

All statistical analyses were performed using Python (version 3.13.5) with the pandas (version 2.2.3), numpy (version 2.2.2), scipy (version 1.15.1), scikit-learn (version 1.6.1), and statsmodels (version 0.14.6) libraries. Statistical significance was defined as a two-sided *p* < 0.05.

### 5.3. Statistical Power Analysis

Given the relatively small sample size of our clinical validation cohort (N = 61 for the primary susceptibility comparison: 35 refined hydrocele cases vs. 26 urolithiasis controls), an a priori power analysis was conducted. Assuming a 1-SD increase in PRS confers an OR of 1.5, statistical power was approximately 18% (α = 0.05) for the logistic regression. For the linear regression, assuming the 5-SNP PRS explains 5% of phenotypic variance (f^2^ = 0.05) alongside four clinical covariates, estimated power was approximately 32% (α = 0.05).

During the preparation of this manuscript, the author(s) used ChatGPT 5.6 (OpenAI), Gemini (Google), and Claude (Anthropic) for language editing, for assistance in developing computational code for genomic data analysis, and for drafting and revising manuscript text in response to reviewer comments. The authors have reviewed and edited all such output and take full responsibility for the content of this publication.

## Figures and Tables

**Table 1:** Participant characteristics (unchanged from the previous submission). Control 1: Prostate cancer patients with bilateral orchiectomy; Stone Control: Urolithiasis patients without prior malignancy or hydrocele history. p1: Hydrocele vs. Control 1; p2: Hydrocele vs. Stone Control. Age and PRS compared by *t*-test; drink and occupation by chi-square test; smoking status (Brinkman Index, BI) by Cochran–Armitage test. Pairwise comparisons were pre-specified and performed without an overall global test.

Variable	Hydrocele	Control 1	Stone Control	p1	p2
Age (years)	65.2 ± 14.0 (n = 47)	69.2 ± 16.1 (n = 35)	60.0 ± 12.1 (n = 26)	0.088	0.294
PRS	−0.162 ± 0.222 (n = 35)	−0.121 ± 0.207 (n = 31)	−0.288 ± 0.187 (n = 26)	0.442	0.020
Drink, yes n (%)	24 (80.0%)	21 (75.0%)	21 (95.5%)	0.313	0.254
Drink, no n (%)	4 (20.0%)	7 (25.0%)	1 (4.5%)		
Smoking, BI 0, n (%)	6 (20.7%)	10 (34.5%)	6 (27.3%)	0.650	0.452
Smoking, BI 1–399, n (%)	4 (13.8%)	2 (6.9%)	6 (27.3%)		
Smoking, BI 400–799, n (%)	12 (41.4%)	8 (27.6%)	4 (18.2%)		
Smoking, BI ≥ 800, n (%)	7 (24.1%)	9 (31.0%)	6 (27.3%)		
Occupation, manual n (%)	12 (42.9%)	13 (48.1%)	11 (50.0%)	0.694	0.891
Occupation, non-manual n (%)	16 (57.1%)	14 (51.9%)	11 (50.0%)		

**Table 2:** Multivariable logistic regression for hydrocele testis susceptibility (unchanged from the previous submission). OR = odds ratio; CI = 95% confidence interval. Continuous and ordinal variables were standardized before analysis. Missing clinical covariate data were imputed using multiple imputation by chained equations (MICE).

Variable (per 1-SD)	Hydrocele > 1 mL vs. Control 1	Hydrocele > 1 mL vs. Stone Control	Hydrocele > 5 mL vs. Stone Control	Hydrocele > 10 mL vs. Stone Control
Age	OR = 0.73 (0.42–1.26), *p* = 0.259	OR = 0.99 (0.55–1.78), *p* = 0.963	OR = 1.05 (0.58–1.92), *p* = 0.865	OR = 1.19 (0.63–2.22), *p* = 0.592
Brinkman index	OR = 1.17 (0.72–1.90), *p* = 0.526	OR = 1.11 (0.64–1.91), *p* = 0.723	OR = 1.14 (0.65–2.00), *p* = 0.655	OR = 1.18 (0.66–2.09), *p* = 0.582
Drink (yes vs. no)	OR = 2.28 (0.56–9.30), *p* = 0.251	OR = 0.53 (0.13–2.09), *p* = 0.366	OR = 0.50 (0.12–2.11), *p* = 0.347	OR = 0.50 (0.11–2.24), *p* = 0.363
Occupation (manual)	OR = 0.54 (0.19–1.51), *p* = 0.237	OR = 0.64 (0.22–1.85), *p* = 0.413	OR = 0.69 (0.23–2.03), *p* = 0.499	OR = 0.94 (0.30–2.91), *p* = 0.912
PRS	OR = 0.78 (0.49–1.25), *p* = 0.309	OR = 2.21 (1.14–4.28), *p* = 0.019	OR = 2.13 (1.09–4.17), *p* = 0.028	OR = 1.98 (1.01–3.88), *p* = 0.048

**Table 3:** Summary of logistic regression models for hydrocele testis susceptibility (unchanged from the previous submission). AUC = area under the receiver operating characteristic curve. “PRS *p*” is the significance of PRS as an independent predictor in the full model; “DeLong *p*” is the significance of the difference in AUC between the base and full models.

Control Group	Case Inclusion Threshold	Base AUC	Full AUC	DeLong *p*	PRS OR (95% CI)	PRS *p*
Control 1 (prostate cancer)	All cases (>1 mL)	0.671	0.696	0.432	0.78 (0.49–1.25)	0.309
Stone Control	All cases (>1 mL)	0.622	0.759	0.052	2.21 (1.14–4.28)	0.019
Stone Control	≥5 mL	0.629	0.764	0.047	2.13 (1.09–4.17)	0.028
Stone Control	≥10 mL	0.645	0.733	0.154	1.98 (1.01–3.88)	0.048

**Table 4:** Multiple linear regression results for hydrocele fluid volume among hydrocele cases only (standardized β).

Variable	All Cases (>1 mL), n = 41	≥5 mL, n = 34	≥10 mL, n = 29
Age	β = 0.027, *p* = 0.854	β = 0.046, *p* = 0.757	β = 0.093, *p* = 0.539
Brinkman index	β = −0.082, *p* = 0.537	β = −0.076, *p* = 0.575	β = −0.061, *p* = 0.656
Drink (yes vs. no)	β = −0.296, *p* = 0.449	β = −0.348, *p* = 0.380	β = −0.457, *p* = 0.265
Occupation (manual)	β = −0.158, *p* = 0.596	β = −0.128, *p* = 0.672	β = −0.024, *p* = 0.939
PRS	β = −0.470, *p* = 0.005	β = −0.538, *p* = 0.004	β = −0.536, *p* = 0.010

**Table 5:** Summary of case-only multiple linear regression models for hydrocele fluid volume.

Case Inclusion	n	Base R^2^	Full R^2^	ANOVA *p*	PRS β	PRS *p*
All cases (>1 mL)	41	0.064	0.257	0.005	−0.470	0.005
≥5 mL	34	0.035	0.286	0.004	−0.538	0.004
≥10 mL	29	0.050	0.293	0.010	−0.536	0.010

**Table 6:** SNPs used for calculation of the PRS (unchanged from the previous submission).

SNP	Position (GRCh37)	Beta	Nearest Gene
rs3748916	2:113984033	−0.2484	*PAX8*
rs2164725	2:121125878	0.0026	*INHBB*
rs1731840	7:155651490	0.1409	*SHH*
rs1087056	10:31395761	0.0017	*ZNF438*
rs11170549	12:53814380	0.0023	*AMHR2*

## Data Availability

The data presented in this study are available on request from the corresponding author, subject to ethical and legal restrictions on participant privacy under IRB approval. The analysis code, including the case-only and 0 mL-control sensitivity analyses referenced in the Discussion, is also available from the corresponding author upon request.

## References

[1] Dagur G., Gandhi J., Suh Y., Weissbart S., Sheynkin Y.R., Smith N.L., Joshi G., Khan S.A. (2017). Classifying Hydroceles of the Pelvis and Groin: An Overview of Etiology, Secondary Complications, Evaluation, and Management. Curr. Urol..

[2] Agre P., King L.S., Yasui M., Guggino W.B., Ottersen O.P., Fujiyoshi Y., Engel A., Nielsen S. (2002). Aquaporin water channels–from atomic structure to clinical medicine. J. Physiol..

[3] Hattori M., Tonooka A., Zaitsu M., Mikami K., Suzue-Yanagisawa A., Uekusa T., Takeuchi T. (2014). Overexpression of Aquaporin 1 in the Tunica Vaginalis May Contribute to Adult-Onset Primary Hydrocele Testis. Adv. Urol..

[4] Hattori-Kato M., Okuno Y., Zaitsu M., Fukuhara H., Nomiya A., Mikami K., Takeuchi T. (2023). Hypomethylation of a CpG Site in the CpG Island of the Aquaporin 1 Gene May Be Involved in the Formation of Adult-Onset Non-communicating Hydrocele Testis. Cureus.

[5] Roberson J.L., Neylan C.J., Judy R., Walker V., Tsao P.S., Damrauer S.M., Maguire L.H. (2024). Critical genes in genitourinary embryogenesis are related to the development of adult hydrocele. Sci. Rep..

[6] Kachuri L., Chatterjee N., Hirbo J., Schaid D.J., Martin I., Kullo I.J., Kenny E.E., Pasaniuc B., Witte J.S., Polygenic Risk Methods in Diverse Populations (PRIMED) Consortium Methods Working Group (2023). Principles and methods for transferring polygenic risk scores across global populations. Nat. Rev. Genet..

[7] Khera A.V., Chaffin M., Aragam K.G., Haas M.E., Roselli C., Choi S.H., Natarajan P., Lander E.S., Lubitz S.A., Ellinor P.T. (2018). Genome-wide polygenic scores for common diseases identify individuals with risk equivalent to monogenic mutations. Nat. Genet..

[8] Torkamani A., Wineinger N.E., Topol E.J. (2018). The personal and clinical utility of polygenic risk scores. Nat. Rev. Genet..

[9] Martin A.R., Kanai M., Kamatani Y., Okada Y., Neale B.M., Daly M.J. (2019). Clinical use of current polygenic risk scores may exacerbate health disparities. Nat. Genet..

[10] Lewis C.M., Vassos E. (2020). Polygenic risk scores: From research tools to clinical instruments. Genome Med..

[11] Shaw A., Gipp J., Bushman W. (2009). The Sonic Hedgehog pathway stimulates prostate tumor growth by paracrine signaling and recapitulates embryonic gene expression in tumor myofibroblasts. Oncogene.

[12] Chaves-Moreira D., Morin P.J., Drapkin R. (2021). Unraveling the Mysteries of PAX8 in Reproductive Tract Cancers. Cancer Res..

[13] Boocock J., Leask M., Okada Y., Matsuo H., Kawamura Y., Shi Y., Li C., Mount D.B., Mandal A.K., Asian Genetic Epidemiology Network (AGEN) Consortium (2020). Genomic dissection of 43 serum urate-associated loci provides multiple insights into molecular mechanisms of urate control. Hum Mol Genet..

[14] Cai N., Verhulst B., Andreassen O.A., Buitelaar J., Edenberg H.J., Hettema J.M., Gandal M., Grotzinger A., Jonas K., Lee P. (2025). Assessment and ascertainment in psychiatric molecular genetics: Challenges and opportunities for cross-disorder research. Mol. Psychiatry.

[15] Takeuchi T., Hattori-Kato M., Okuno Y., Zaitsu M., Azuma T. (2022). Genome-Wide Association Study Adjusted for Occupational and Environmental Factors for Bladder Cancer Susceptibility. Genes.

[16] Choi S.W., Mak T.S.-H., O’rEilly P.F. (2020). Tutorial: A guide to performing polygenic risk score analyses. Nat. Protoc..

